# How Does *In Vitro* Digestion Change the Amount of Phenolics in *Morus alba* L. Leaf? Analysis of Preparations and Infusions

**DOI:** 10.3390/metabo14010031

**Published:** 2024-01-01

**Authors:** Monika Przeor

**Affiliations:** Department of Gastronomy Sciences and Functional Foods, Poznań University of Life Sciences, Wojska Polskiego 31, 60-624 Poznań, Poland; monika.przeor@up.poznan.pl; Tel.: +48-61-848-7328

**Keywords:** white mulberry, digestion, phenolic acids, flavonols, processing, conditioning

## Abstract

The application of *Morus alba* L. in traditional oriental medicine and cuisine has resulted in numerous studies on its health-promoting effects. However, if the process is not monitored by the manufacturers, the processing of the leaves alters the obtained health-promoting properties and results in different health qualities in the final composition of dietary supplements. This article aims to analyze changes (using the HPLC/DAD method) in the proposed conditioned mulberry leaves in terms of key compounds (phenolic acids and flavonols) responsible for antioxidant activity after being digested in *in vitro* conditions. The analyzed material was leaves of white mulberry (*Morus alba* L.) cv. Żółwińska wielkolistna, conditioned (1–4 h) and non-conditioned. The conditioning process of mulberry proposed here, e.g., for industry production, resulted in variable transformations of polyphenols during *in vitro* digestion. For many polyphenols, especially those shown in the highest amounts, significant correlations were found between their content and conditioning, as well as the stage of digestion. In the case of mulberry infusions, the amounts of individual polyphenols were several times lower than in the preparations, which was due to the degree of dilution. Their amounts tended to decrease in the course of digestion. Taking this into account, it seems justified to continue research on the *in vivo* bioavailability of bioactive components from conditioned *Morus alba* L. leaves.

## 1. Introduction

White mulberry (*Morus alba* L.) is one of the plants that can grow in various climatic conditions. Its common application in traditional oriental medicine has resulted in numerous studies on its health-promoting properties [1,2,3]. The leaves of white mulberry are commonly known and used in dried form for infusions. However, novel application methods are being sought for leaf extracts, e.g., as an ingredient in bread, dairy products, or candies [4,5,6,7,8,9], despite the legal difficulties related to adding *Morus* leaves to food products in some regions, e.g., the European Union. Mulberry leaves differ in chemical composition, including in terms of polyphenols, depending on the cultivar or species used and growing conditions, which results in diverse functional properties [10]. What is more, the processing of the leaves may also modify their properties due to the changes occurring during the treatment processes.

Polyphenols are currently becoming more widely available as components of dietary supplements or functional food products. Despite the increasing body of evidence regarding the pharmacological efficacy of their effects on human health, there is a parallel concern about their actual activity in living organisms. What is meant here is, first of all, the relationship between chemical structure and intestinal absorption, metabolism, and bioavailability of polyphenols [11]. This is because it turns out that studies conducted *in vitro* cannot fully reproduce *in vivo* conditions. The process of digestion of polyphenols in a living organism is complex and depends on many factors, including the concentration of the components and the matrix in which they are delivered, the linkages with other structures, the profile of the gastrointestinal microflora (individually variable, depending, inter alia, on health status and diet), the current activity of digestive enzymes, etc. [12,13]. 

Therefore, *in vitro* digestion is used at certain stages of the analysis of individual raw materials. It is pointed out that only *in vitro* studies use experimentally standard microbiological material, the selected specific enzymes, and accurately described environmental conditions [14].

The studies on the digestibility of polyphenols use different process conditions, depending on the character and concentration of the material, duration of each digestion step, presence and/or composition of intestinal microorganisms, etc. [15,16,17].

Moreover, polyphenols play an essential role in many important diet-related mechanisms, e.g., by modifying glucose metabolism in diabetes mellitus [2,18], and even on the gut-brain axis [19]. However, the amount of each phenolic compound can vary under different conditions, both at harvest and during processing [20,21,22].

**Figure 1:**
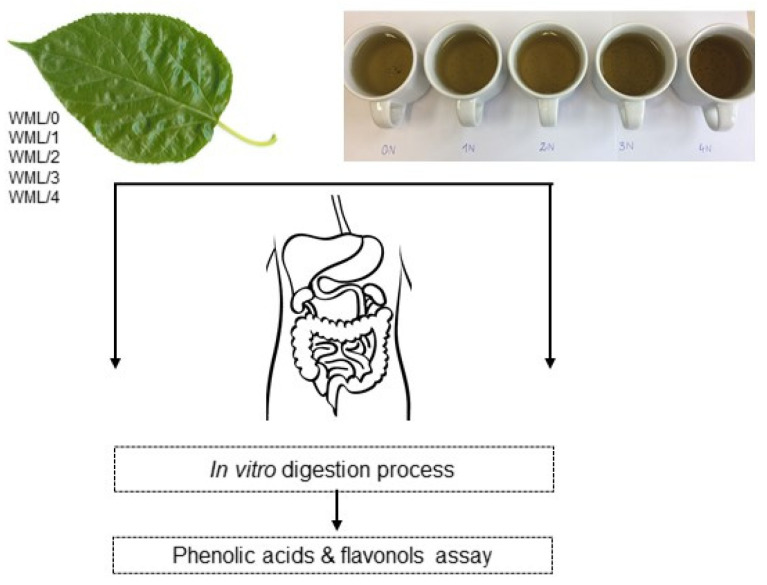
Scheme of analyses conducted for leaf preparations and infusions from leaf preparations of *Morus alba*.

The authors have already proposed [23] an interesting processing method—a simple conditioning processing of *Morus alba* leaves. However, that study did not address the digestibility of selected biologically active compounds. This paper aims to fill this important gap based on European material. It also constitutes the next stage of chemical analysis of conditioned mulberry leaves, which involves the determination of key compounds (phenolic acids and flavonols) following *in vitro* digestion, taking into account the specific stages and the chemical changes occurring at those stages (Figure 1).

This research has led to a better understanding of the changes that occur in the *Morus alba* leaves during technological processing. This work contributes a considerable amount of information for future optimization of white mulberry leaf processing, for example, in the production of dietary supplements or as a food ingredient. Furthermore, the results provide a further argument for improving the processing of mulberry leaves by processing companies to get a valuable, high-quality intermediate product beneficial for consumers’ health.

## 2. Materials and Methods

### 2.1. Material

The analyzed material was the leaves of white mulberry (*Morus alba* L.) cv. Żółwińska wielkolistna. The leaves were collected at the Silk Moth Breeding and Mulberry Farming Research Group of the Institute of Natural Fibers and Medicinal Plants located in Pętkowo, Greater Poland Voivodeship, Poland (DMS: 52°12′13.448″ N 17°15′8.607″ E). The Polish white mulberry cultivar Żółwińska wielkolistna was selected in the 1950s at the Natural Silk Research Facility in Żółwin near Milanówek, Poland, for silkworm feeding by the Head of Facility—Henryk Witaczak, creator of Polish silk. By the decision of the director of the Institute of Natural Fibres in Poznań, the collection of this plant was transferred in 2004 to the Research Farm in Pętkowo. Official data from the Institute state that the Polish variety of white mulberry is characterized by its very large leaf blades and rapid growth, as it was selected for the breeding of the mulberry silkworm. These plantings started a pro-ecological plantation of the Polish mulberry variety, which is unique throughout the country. The Institute of Natural Fibers and Medicinal Plants in Poznań was granted exclusive rights to the Żółwińska wielkolistna variety [24].

### 2.2. Conditioning Process of Leaves

The mulberry leaves were twisted and blended using a cutting-edge electric garden shredder (Stihl, Viking GE 103, Dieburg, Germany). The leaves were divided into five groups. Four of all groups were collected in 10–15 cm high piles on wooden trays with sieve bottoms and then conditioned in the drying chamber in four different time ranges:

- 1 h–conditioned for 1 h;
- 2 h–conditioned for 2 h;
- 3 h–conditioned for 3 h;
- 4 h–conditioned for 4 h.

The conditioning process was performed at a controlled temperature of 32.0 °C ± 3.0. Meanwhile, a control sample of leaves not subjected to conditioning was prepared (0 h).

Subsequently, leaves were dried in a belt-drum dryer at a temperature of ~90 °C at the input and ~60 °C at the output. Dried leaves of *M. alba* were packed into 50 L paper bags and stored under cooling conditions with low humidity. Before the extraction, the dried leaves were ground in a cutting mill (Retsch, model GM200, velocity 4000 rpm by 15 s) and then stored at ambient temperature in transparent plastic (PE) string bags with a capacity of 500 mL.

### 2.3. Extraction Process

To perform further analyses, extracts were prepared by double maceration (100 °C, 15 min each) of mulberry leaves (10.0 g ± 0.2) with water (1st—100 mL, 2nd–40 mL), followed by filtration with a Büchner funnel in duplicates, according to the protocol developed by Przeor et al. (2020) [23].

### 2.4. Infusions Preparation

Infusions of white mulberry leaves were obtained using the method recommended by a producer of white mulberry leaf infusions available on the Polish market. Infusions were prepared directly before use. New infusions were made for each test repetition.

The infusions were encoded as follows:

- 0N–infusion from non-conditioned leaves;
- 1N–infusion from leaves conditioned for 1 h;
- 2N–infusion from leaves conditioned for 2 h;
- 3N–infusion from leaves conditioned for 3 h;
- 4N–infusion from leaves conditioned for 4 h.

The entire amount of 2.0 ± 0.1 g of dried leaves was transferred to a 300 mL flask, and 250 mL of boiling tap water was added. Next, the flask was covered with foil, and the start time of the infusion process was recorded. After 15 min, the foil was removed, the infusion was stirred in a figure-of-eight pattern for 15 s, and then filtered through filter paper (Whatman No. 1, 1–11 μm) placed in a Büchner funnel. The fresh-brewed and filtered infusions were directly used in *in vitro* digestion.

### 2.5. In vitro Simulation of Digestion Process

The *in vitro* digestion study involved the preparation of both the leaves and the infusion made of them. The simulation was performed with the use of a static *in vitro* digestive model. The process occurred in a BIOSTAT B Plus (Sartorius Stedim Biotech, Göttingen, Germany) laboratory bioreactor with a sealed 1 L mono tank, surrounded by a water jacket, maintaining the set process temperature (37 °C) to imitate conditions in the human digestive tract. The bioreactor had four peristatic pumps dosing the reactants into the monotank: two pumps (with 1 M HCl and 1 M NaOH) keeping the acidity of the process, and two other pumps dosing digesting enzymes and bile acid salts (pepsin in 0.1 M HCl and a mixture of pancreatin and bile acid salts in 0.1 M NaHCO_3_) (Table 1).

The bioreactor software enabled changing conditions inside the tank depending on the simulated digestion stage. Samples were added through a special hole in the tank shell, and the inside paddle stirrer dispersed the sample homogenously in the entire volume of the tank. The intestinal microflora was introduced into the BHI solution. Sampling was done sequentially: at the beginning of the stomach stage (A), at the end of the stomach stage (B), at the end of the duodenum stage (C), at the beginning of the small intestine (D), at the end of the small intestine (E), and at the end of the large intestine (F). Sampling was performed aseptically (in triplicates) using a system in the lid of the collection vessel that prevents contamination of the digested material solution. Samples were stored in frozen form.

To ensure aseptic conditions, bioreactor components, vessels, solutions, and small equipment were sterilized each time before the experiment using an autoclave (ASVE 400 × 600, SMS) or a laminar chamber (Safe 2020, ThermoScientific, Burladingen, Germany), while a 72% ethanol solution was additionally applied to external surfaces during the digestion process. The device was stabilized each time, and the electrode was calibrated.

### 2.6. High-Performance Liquid Chromatography Assay–Phenolic Acids and Flavonols

The content of phenolic acids and flavonols in the samples was determined using high-performance liquid chromatography with a diode array detector (HPLC/DAD) technique (Agilent Infinity 1290, Santa Clara, CA, USA). A Zorbax SB C18 column (150 mm × 3.9 mm ID, 5 µm) (Agilent Technology, Santa Clara, CA, USA) was used for separation, based on the method described by Przeor et al. (2020) [23].

Detection of separated phenolic acids was performed at λ = 260 nm: gallic acid (GAL) (y = 54.013x + 143.85), protocatechuic acid (PRO) (y = 191.07x + 82.424), 4-hydroxybenzoic acid (HYD) (y = 301.61x + 15.069), vanillic acid (VAN) (y = 188.56x + 9.42), caffeic acid (CAF) (y = 48.406x + 11.419), and at λ = 310 nm: chlorogenic acid (CHL) (y = 277.55x + 51.95), syringic acid (SYR) (y = 107.48x + 23.398), *p*-coumaric acid (COU) (y = 428.009x + 2.376), ferulic acid (FER) (y = 270.86x + 14.292), sinapic acid (SIN) (y = 227.16x + 12.951).

Flavonols were detected at λ = 370 nm: rutin (RUT) (y = 0.656x − 1.096), isoquercitrin (ISQ) (y = 1.099x + 0.929), quercetin 3-O-(6″-*O*-malonyl)-β-D-glucoside (MAL) (y = 0.794x + 0.952), astragalin (AST) (y = 0.901x − 1.744), myricetin (MYR) (y = 1.685x − 4.267), quercetin (QUE) (y = 2.121x − 8.568), kaempferol (KEM) (y = 2.284x − 6.032), isorhamnetin (ISR) (y = 2.317x − 24.833). Tests were performed in triplicate. The external standard method was used for the quantitative assay. Results were expressed as μg × mL^−1^ of the analyzed material.

### 2.7. Statistical Analysis

Statistical analysis of the results was performed in Statistica 13 (StatSoft). The first step was to perform tests to check the normality of the distribution with the Shapiro–Wilk test. Multiple comparisons (post-hoc tests), as well as one-factor or two-factor analysis of variance, were used to determine statistically significant differences between mean scores in a given set. A significance level characteristic of the natural sciences of α = 0.05 was used.

### 2.8. Reagents

Standards for phenolic acids and flavonols were purchased from Sigma–Aldrich, Taufkirchen, Germany. Reagents for the simulated digestion process were purchased from VWR, Poland, and Sigma–Aldrich, Taufkirchen, Germany. Other chemicals of analytical or chromatographic grade were purchased from POCH, Gliwice, Poland, or Merck, Darmstadt, Germany.

## 3. Results

### 3.1. Simulated Digestion of White Mulberry Leaf Preparations

**Figure 2:**
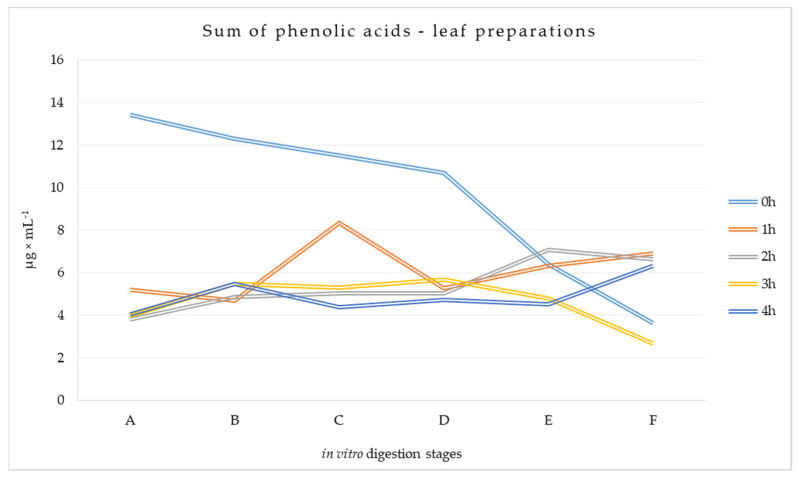
Total phenolic acid content at different stages of simulated digestion of conditioned and non-conditioned *Morus alba* L. leaf preparations [μg × mL^−1^]. A—sampling at the beginning of the stomach stage, B—sampling at the end of the stomach stage, C—sampling at the end of the duodenum stage, D—sampling at the beginning of the small intestine, E—sampling at the end of the small intestine, F—sampling at the end of the large intestine.

**Figure 3:**
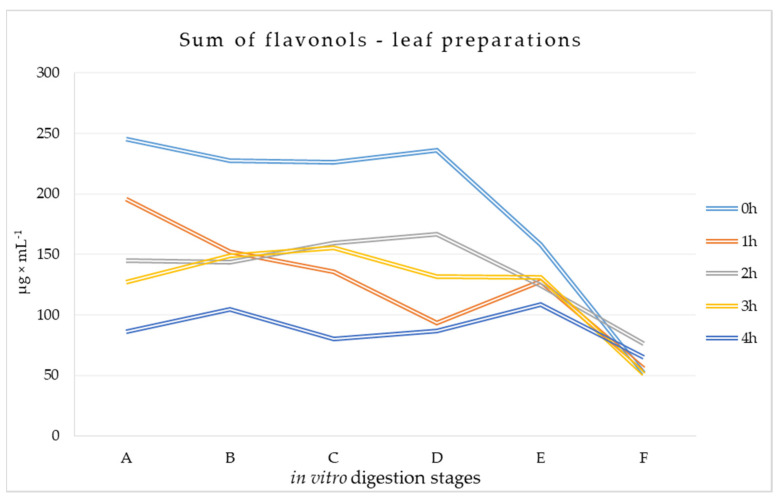
Total flavonols at different stages of the simulated digestion of conditioned and non-conditioned *Morus alba* L. leaf preparations [μg × mL^−1^]. A—sampling at the beginning of the stomach stage, B—sampling at the end of the stomach stage, C—sampling at the end of the duodenum stage, D—sampling at the beginning of the small intestine, E—sampling at the end of the small intestine, F–sampling at the end of the large intestine.

According to the research model, the digestion process of mulberry preparations was simulated using a model of the human digestive tract. The preparations were subjected to established, reproducible transformations characteristic of successive stages of digestion from the stomach to the large intestine. The results are summarized below in Figure 2 and Figure 3.

#### 3.1.1. Phenolic Acid Content at Different Stages of Digestion of Leaf Preparations

Quantitative changes in different stages of the simulated gastrointestinal tract were also evident for phenolic acids. With regard to retention times and UV-Vis spectra, ten phenolic acids were identified. Quantitatively, the individual compounds are shown in Table S1. Gallic acid (2.078–7.964 μg × mL × mL^−1^) predominated at all stages of the digestion process, while chlorogenic acid (0.096–2.564 μg × mL^−1^) and caffeic acid (0.082–2.181 μg × mL^−1^) were also high. It was observed that as digestion progressed, the amount of gallic, 4-hydroxybenzoic, vanillic, and caffeic acids showed an increasing trend, with few exceptions (GAL 2 h and 4 h, CAF 4 h, VAN 3 h). In the case of protocatechuic, chlorogenic, syringic, and ferulic acids, in the vast majority, their amount was reduced to a level lower than that found at stages A–2-fold (FER 4 h), 6-fold (PRO 2 h), 12-fold (CHL 4 h), or 30-fold (SYR 0 h). In addition, the composition of the analyzed material resulting from the digestion of leaves conditioned for one, two, and three hours in terms of the content of individual phenolic acids was very similar to each other. The significance of the differences in the Tukey test between the different stages of digestion for each phenolic acid is indicated by the letters above the bars of the mean values.

In the analyzed material obtained during the simulated digestion of various leaf preparations, the 0 h variant had the highest total phenolic acid content (Figure 2). This was evident at stages A, B, C, and D, where up to three times more phenolic acids were recorded compared to the material obtained from conditioned leaves. Preparations from conditioned leaves were not very different from one another, although, at stage C (duodenum) of the 1 h variant, the amount of phenolic acids almost doubled compared to the sample taken at stage B (stomach). As a result of colon conditions, the amount of phenolic acids in the analyzed material decreased in three samples (1 h, 2 h, and 4 h). 

The content of individual phenolic acids at different stages of simulated digestion did not have a normal distribution in the Shapiro–Wilk test (*p* < 0.05). Therefore, the results were analyzed using nonparametric tests (Table 2). A very high correlation was found between the content of syringic acid and the digestion stage. Also, there was a high correlation between the content of protocatechuic acid and 4-hydroxybenzoic acid, the time of conditioning, and the stage of digestion (*p* ≤ 0.05). A moderate (gamma correlation and Kendall’s τ correlation) or strong (Spearman’s rank order correlation) correlation was observed for chlorogenic acid content compared to subsequent digestion stages, and a moderate correlation was found between vanillic and sinapic acid content and digestion stages. In addition, the content of both acids (VAN and SIN) correlated weakly, but statistically significantly, with conditioning time. The content of caffeic acid was also weakly correlated (*p* ≤ 0.05) with the conditioning time of the leaves used in the study.

#### 3.1.2. Flavonols Content at Different Stages of Digestion of Leaf Preparations

The *in vitro* digestion process resulted in fairly regular changes in the amounts of individual flavonols (Table S2). Starting from the small intestine stage (D or E), the amounts analyzed were gradually reduced, with the greatest reduction occurring at the terminal stage of the large intestine. For many flavonols, the decrease in their content throughout the process was preceded by an initial increase in their content at the A–D stages. Among the identified flavonols, rutin and quercetin 3-(6-malonyl)-glucoside were found in the highest amounts, 33.394–103.883 μg × mL^−1^ and 30.610–82.117 μg × mL^−1^, respectively, at the beginning of the digestion process. In addition, lower amounts of isoquercitrin (11.001–32.602 μg × mL^−1^), astragalin (4.581–11.976 μg × mL^−1^), and myricetin (5.352–13.919 μg × mL^−1^) were found. The contents of quercetin, kaempferol, and isorhamnetin were below 1 μg × mL^−1^ at all digestion stages. The smallest variations in content between stages were recorded for isorhamnetin in all analyzed variants, astragalin in 1 h, 2 h, and 3 h variants, and quercetin in 0 h, 1 h, and 3 h variants. It was noted that WML preparations (4 h) often had the lowest content of individual flavonols. Moreover, among the preparations made from conditioned leaves, the 2 h or 3 h variants had the highest content of the analyzed flavonols.

Considering the total amount of flavonols, the analyzed digested material resulting from the transformation of WML preparations conditioned for four hours had the lowest amount of flavonols of all variants (Figure 3). Variant 0 h proved to be the richest, with total flavonols almost twice as high as in variant 3 h, as well as 60–70% higher than in variant 2 h and 25–50% higher than in variant 1 h, especially at the gastric stages of the digestion process (A and B). The amounts of flavonols found at stage F differed between variants by a maximum of 48%. The greatest quantitative losses of total flavonols were observed at stage F, amounting to 39–67%.

The statistical analysis revealed significant linear correlations between the content of some flavonols (ISO, MAL, and MYR) and the stage of digestion, as well as the time of conditioning, although the latter was significant only for isoquercitrin (Table 3). In addition, it was found that the determined content of astragalin and quercetin correlated significantly with the conditioning time of the mulberry leaves used for preparation in non-parametric tests, although not at a high level (astragalin 0.219–0.273 and quercetin 0.349–0.462). A statistically significant effect on the content of rutin, quercetin, and isorhamnetin was observed for the stages of the simulated digestion process. 

### 3.2. Simulated Digestion of White Mulberry Leaf Infusions

#### 3.2.1. Phenolic Acids Content at Different Stages of the Digestion Process of Mulberry Infusions

Chromatographic analysis showed that gallic acid (0.190–0.601 μg × mL^−1^ of analyzed material at the beginning of the process) predominated in the leaf infusions targeted for digestion (Table S3). Its amounts gradually increased as digestion progressed, reaching values ranging from 0.791 μg × mL^−1^ in the case of 4N to 3.490 μg × mL^−1^ in the case of 3N at the end of the large intestine (F). The lowest differences in results in this range were observed for infusions of leaves that were not conditioned, leaves conditioned for one hour, and leaves conditioned for two hours.

Caffeic acid was present in the infusions at various levels, and a decreasing tendency was observed in subsequent infusions with the conditioning time of the WML from which they were made. The weakest effect of varying conditions of the digestion process on the determined content of caffeic acid was found in infusions from non-conditioned leaves (0.511–0.614 μg × mL^−1^). A similar trend was observed for chlorogenic acid. The highest amounts of protocatechuic acid were identified for infusions of non-conditioned leaves at all stages of the digestion process.

**Figure 4:**
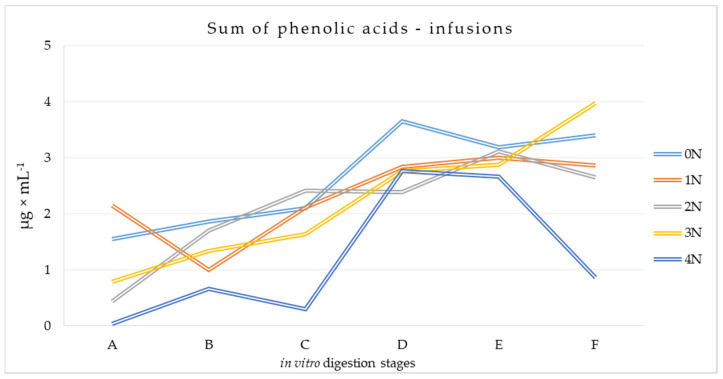
Total phenolic acid content at different stages of the simulated digestion process of infusions of conditioned and non-conditioned *Morus alba* L. leaves [μg × mL^−1^]. A—sampling at the beginning of the stomach stage, B—sampling at the end of the stomach stage, C—sampling at the end of the duodenum stage, D—sampling at the beginning of the small intestine, E—sampling at the end of the small intestine, F—sampling at the end of the large intestine.

The 4N Infusion variant was found to be the least susceptible to *in vitro* digestion. Several times higher amounts of 4-hydroxybenzoic acid were determined at stage F compared to stage A for all infusions. Similar quantitative changes were recorded for vanillic acid in the 0N, 3N, and 4N variants. At the intestinal stage, syringic acid was generally not observed, while sinapic acid was identified only in infusions of non-conditioned leaves. Ferulic acid was found to be fairly stable during the digestion process in all analyzed infusions. The sum of identified phenolic acids after *in vitro* digestion is shown in Figure 4. It was confirmed that the content of labeled total phenolic acids at the beginning of the process was highest for 1N and 0N infusions. As the digestion process progressed, the amounts were generally higher, in a few cases decreasing at stage F (infusion 1N by 5%, 2N by 15%, 4N by 68%).

The compiled results did not have a normal distribution according to the Shapiro–Wilk test (*p* < 0.05), so they were subjected to statistical analysis with non-parametric tests. Correlations between the two main independent variables (conditioning and digestion stage) and the dependent variable (the content of a particular phenolic acid in the sample) were determined using three tests (Table 4). For all phenolic acids, a negative correlation was found between conditioning time and the determined amounts of these polyphenols. For such phenolic acids as protocatechuic, chlorogenic, caffeic, syringic, *p-*coumaric, ferulic, and sinapic acids, the correlations in Spearman’s rank-order test showed moderate strength. Ferulic (−0.818) and caffeic acids (−0.813) showed by far the highest correlation in this regard. A weak correlation was observed for gallic acid, 4-hydroxybenzoic acid, and vanillic acid. On the other hand, when analyzing the effect of the digestion stage on the determined content of gallic acid, 4-hydroxybenzoic acid, vanillic acid, and caffeic acid, positive correlations were noted in Spearman’s rank order test (0.767, 0.247, 0.127, and 0.189, respectively).

#### 3.2.2. Flavonols Content at Different Stages of the Digestion Process of Mulberry Infusions

The amounts of flavonols were determined in the digested leaf infusions (Table S4). Out of the seven identified flavonols, rutin and quercetin 3-(6-malonyl)-glucoside predominated in the samples, confirming previous findings in leaf preparations. It was observed that the amount of compounds present in the samples depended on the stage of the simulated digestion process and the type of infusion. The most abundant of the flavonols tested was rutin, whose content at the different stages of the digestion process was generally highest in the second stage of digestion (7.437–18.094 μg × mL^−1^ of digestive content), and then it decreased. Considerable amounts of quercetin 3-(6-malonyl)-glucoside were also recorded, the content of which decreased during the digestion process of the infusions, most in the 0N and 3N variants. In the case of isoquercitrin, astragalin, and myricetin, similar decreases were observed, although at a lower quantitative level. Slightly smaller differences at successive stages of the digestion process were observed for quercetin, except for infusions of leaves conditioned for one hour, and for kaempferol. In all variants, the proportion of quercetin (0.220–0.724 μg × mL^−1^ of digestive content) and kaempferol (0.132–0.327 μg × mL^−1^ of digestive content) was the lowest. Isorhamnetin was not found in any of the leaf infusions subjected to simulated digestion. In addition, leaf infusions conditioned for four hours contained lower amounts of individual flavonols than the other variants targeted for *in vitro* digestion.

**Figure 5:**
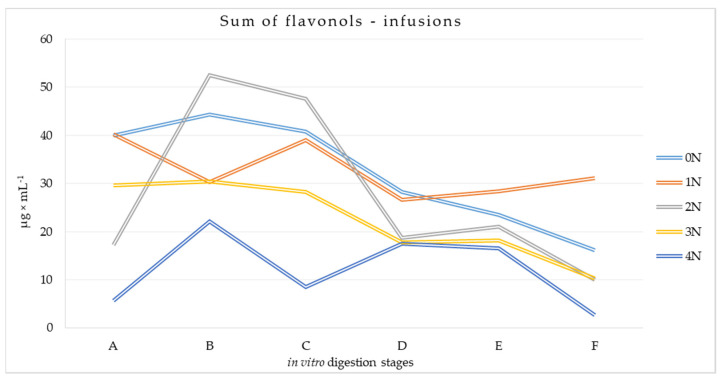
Total flavonols at different stages of the simulated digestion process of infusions of conditioned and non-conditioned Morus alba L. leaves [μg × mL^−1^]. A—sampling at the beginning of the stomach stage, B—sampling at the end of the stomach stage, C—sampling at the end of the duodenum stage, D—sampling at the beginning of the small intestine, E—sampling at the end of the small intestine, F—sampling at the end of the large intestine.

The total content of flavonols was variable along the different sections of the simulated gastrointestinal tract (Figure 5). Stomach conditions favored an increase in flavonols. A subsequent progressive increase in environmental pH generally led to a decrease in the amount of flavonols. Subjecting the infusion samples to conditions specific to the large intestine resulted in a decrease in their content at the end of the process by at least ⅓ (0N).

The statistical analysis showed that there was a negative correlation between conditioning time and the determined content of individual flavonols (Table 5). The obtained absolute values in Spearman’s rank-order correlation test in the range of 0.412–0.639 for conditioning time indicated that the relationship was moderate. The same was true for the effect of successive digestion stages on the content of rutin, isoquercitrin, quercetin 3-(6-malonyl)-glucoside, and quercetin. For kaempferol, astragalin, and myricetin, the correlation strength of the digestion stage and flavonol content was found to be weak, due to the │r│values being in the range of 0.2–0.4. In other tests, the values for correlation strength were lower. All the results were statistically significant.

Table 6 summarizes the correlation coefficients between the indices obtained for the digested preparations and those obtained for the digested infusions. The results obtained from the simulated digestion of the preparations and infusions correlated significantly with each other in terms of flavonol content.

## 4. Discussion

Phenolic acids and flavonols belong to the group of polyphenols, whose presence determines antioxidant activity. This study also analyzed benzoic and cinnamic acid derivatives, the amounts of which in leaf preparations increased during conditioning.

As already shown, leaf preparations produced in a laboratory and conditioned for three hours contained the most total phenolic acids and flavonols [23], while preparations from non-conditioned leaves contained 70–80% less. On the other hand, on the semi-technical scale, the content of total phenolic acids and flavonols increased only after two hours of conditioning and then decreased. Chlorogenic acid and caffeic acid were the dominant acids in the leaf preparations. The dominant flavonols in all preparations were rutin, isoquercitrin, astragalin, and quercetin 3-(6-malonyl)-glucoside, similar to those in Korean leaves [25]. Lee and Choi (2012) [25] found rutin in the amount of 46.46–161.76 mg × 100 g^−1^ d.m., isoquercitrin in the amount of 30.40–66.81 mg × 100 g^−1^ d.m., and astragalin in the amount of 24.41–25.18 mg × 100 g^−1^ d.m. of leaves harvested in May. The flavonol specific for mulberry leaves—quercetin 3-(6-malonyl)-glucoside—is a significant bioactive component with anti-atherogenic and anti-hyperglycemic effects [26,27]. The content of quercetin 3-(6-malonyl)-glucoside increased by 31% only in the course of two-hour conditioning. The changes in the raw material in the course of longer (three- and four-hour) conditioning resulted in a significant reduction, with some increase in quercetin content. Thus, as a result of the activity of endogenous esterases released during leaf crushing, hydrolysis of this flavonol took place, resulting in the release of the quercetin aglycone. It is suspected that up to 50% of the anti-diabetic effect of mulberry extracts may depend on the presence of the two main polyphenols of mulberry leaves, i.e., chlorogenic acid and rutin [28]. Chlorogenic acid is credited with the ability to attenuate glycogenolysis and reduce glucose absorption, as well as having strong antioxidant properties [29,30]. On the other hand, rutin protects against tumorigenesis and inhibits peroxidation of the LDL fraction of cholesterol [31].

The digestion process of the produced leaf preparations and their infusions was simulated *in vitro*. A multi-element gastrointestinal model—a mixture of bioactive compounds suspended in a plant matrix—was used to digest leaf preparations. A sequential arrangement was used, in which each successive step followed immediately the previous one at identical time intervals and in possibly identical experimental conditions. Thus, the biochemical conditions present in the mouth, stomach, and intestines were reproduced in the *in vitro* conditions of the bioreactor.

The first step of digestion was to simulate oral conditions, which was achieved by holding the sample in a bioreactor bowl for 10 min at 37 °C to homogenize it. Low pH conditions were then created, and pepsin was dosed, mimicking the gastric section of the GI tract. The study found that passing the samples through stages simulating the oral cavity and the stomach did not always result in a significant quantitative change in the concentration of phenolic acids and flavonols. Different trends were observed between formulations—polyphenol content decreased for the 0 h and 1 h formulations and increased for the 2 h, 3 h, and 4 h variants. In the case of infusions, polyphenol content decreased only in the variant conditioned for one hour. Such results of increasing or decreasing polyphenol concentrations at the initial stages of the simulated digestion process are supported by the literature. Some studies have pointed to the loss of polyphenols during the stages of oral and gastric conditions [32,33], while other authors have emphasized the stability of compounds during the passage through these stages. This was explained by the too-short exposure of the samples to the acidic environment of the stomach, which did not involve hydrolysis or the release of polyphenols from cellular structures. In addition, it is speculated that low pH has a protective effect on polyphenol structures [34].

After incubation in simulated stomach conditions, the intestinal stage was conducted in the bioreactor. The greatest decreases in phenolic acids and flavonols were observed after the intestinal conditions were applied to both preparations and infusions of the leaves. This is fully justified because polyphenols that reach the colon are intensively processed into a simpler form by the microflora residing there. Their presence can also affect the growth of major strains of intestinal bacteria [35]. It is increasingly emphasized that the antioxidant capacity of plant-derived foods is evidenced not only by the content of polyphenols but also by the activity of phenolic metabolites of bacterial origin, whose high concentrations are recorded precisely within the colon [36].

Polyphenols are generally poorly absorbed during digestion, as they are converted through the action of digestive enzymes and intestinal microflora to lower molecular-weight compounds. Animal and human studies have shown this to be the case for many polyphenols, including chlorogenic acid, caffeic acid, ferulic acid, and rutin [37].

As a result of the simulated digestion process, a significant reduction in chlorogenic acid content was observed in the digested leaf preparations, except for the 4 h preparation. A reduction in its content of at least 53% at the end of the intestine was recorded for conditioned leaves and 99% in the preparation from non-conditioned leaves. Losses of chlorogenic acid in a study by Siracusa et al. [33] amounted to 58% after passage through the simulated stomach and 95% after passage through the simulated intestines. Caffeic acid content after the passage of the non-conditioned samples through the simulated intestines decreased by 73% compared to the stomach stage. However, in the conditioned variants, an increase in the content of this acid was observed in the subsequent stages of the digestion of the preparations. For chemically pure caffeic acid, Tagliazucchi et al. (2010) [38] observed a ca. 25% reduction in its content and over 43% in gallic acid content at the intestinal stage.

In the intestines, as a result of bacterial esterase activity, the ester bond of chlorogenic acid is cleaved with the release of caffeic acid and chinoic acid. This generates caffeic acid, and later, after dehydroxylation, also 3-(3-hydroxyphenyl)-propionic acid [39]. The amount of caffeic acid can therefore be considered an indicator of the progress of chlorogenic acid transformation in the preparation. In the analyzed preparations, caffeic acid was present in all samples, and the increasing content of caffeic acid at successive stages of digestion was attributed to the hydrolysis of chlorogenic acid, which was manifested by a decrease in its amount during the process of simulated digestion. During the digestion process, caffeic acid can be *O*-methylated and converted to ferulic and dihydroferulic acids, and further to vanillic acid [39]. In addition, the intestinal metabolism of caffeic acid leads to the formation of dihydrocaffeic acid and *m*-coumaric acid [39]. The confirmation of all these transformations, starting with chlorogenic acid and continuing with caffeic acid, was the presence of ferulic, isoferulic, dihydroferulic, and vanillic acids in the urine of people consuming coffee enriched with chlorogenic acid [37]. Of those mentioned, ferulic acid and vanillic acid were identified in the preparations discussed. In the experiment conducted, a steady reduction in the amount of ferulic acid in the digestive content and an increase in vanillic acid were observed. In a study by Ekbatan et al. (2016) [36], vanillic acid was detected in the transverse colon and descending colon, i.e., the terminal sections of the large intestine. In our study, the same correlation with a reduction in ferulic acid content was observed.

Protocatechuic acid content decreased with the progression of simulated digestion in all preparations, especially in the last section of the intestine, by 56–85%. Protocatechuic acid can be synthesized from caffeic acid as a result of microflora activity in the colon [36] or from quercetin [39]. Such transformations, however, were not dominant, as the amounts of caffeic acid and quercetin in conditioned preparations did not decrease during digestion. Also, the processes of quercetin release from more complex structures or other flavonols may have prevailed over the processes of degradation.

The content of *p*-coumaric acid increased after digestion in the stomach and duodenum in the case of non-digested preparations, conditioned for two and three hours, followed by a reduction after simulating intestinal conditions. Ekbatan et al. (2016) [36] observed an even greater reduction in the amount of coumaric acid than was observed in our experiment.

In this study, a decrease in phenolic acid content was mostly observed in the course of digestion, with the highest in the intestinal section. Such a reduction was also observed by other researchers [40], who found a reduction of phenolic acids of ca. 36–46%. The reduction in the amount of phenolic acids may have been associated with changes in pH and the presence of bile acid salts in the intestine, leading to the precipitation of phenolic acids. In addition, the instability of caffeoylquinic acids in aqueous solutions may have contributed to the observed losses [41].

Among the flavonols, rutin, isoquercitrin, astragalin, and quercetin 3-(6-malonyl)-glucoside predominated. During the simulated digestion process, it was found that the amount of rutin in the digestive content gradually decreased, both during the digestion of preparations and infusions. The effect of acidic gastric conditions was a reduction of ~3–15%, while after the application of intestinal conditions, the reduction was by 61–99%. In the digestive content obtained after the digestion of nondigested preparations at the intestinal stage, rutin was not found, as in a study by Siracusa et al. (2012) [33]. Rutin, or quercetin 3-rutinoside, is a flavonoid glycoside. Its presence is believed to determine the health-promoting properties of the raw material. Due to the metabolism of gut bacteria, rutin and quercetin can be converted to 3,4-dihydrophenylacetic acid, a dopamine neurotransmitter with anti-inflammatory and cardioprotective properties. However, in the absence of free radicals, this acid inhibits cellular respiration in brain mitochondria, contributing to the neurodegeneration associated with Parkinson’s disease [42].

In the course of bacterial decomposition of rutin in the gastrointestinal tract, quercetin is formed, which is quantified in the content of digested preparations and infusions. In the present study, the content of quercetin in the digestive content gradually increased by up to 144% until stage E, i.e., the terminal portion of the small intestine, or remained at a level similar to the earlier stages of digestion. Only after passing to the colon did its amount decrease, in the range of 23–70% depending on the sample variant and in the range of 1–63% depending on the infusion variant.

In a study by Tagliazucchi et al. (2010) [38], the reduction in quercetin was 5.8% after the intestinal stage. Ekbatan et al. (2016) [36] identified quercetin in their study only in the ascending part of the colon, probably released from complex chemical compounds. The increase in the content of quercetin was probably related to the breakdown of other flavonols in whose structure it is found, i.e., quercetin 3-(6-malonyl)-glucoside, rutin, isoquercetin (3-β-D-glucoside of quercetin), and isorhamnetin (3-methylquercetin). The content of these flavonols gradually decreased over the course of the simulated digestion.

As with quercetin, in the digested preparations, astragalin content also increased in the simulated small intestine. However, after implementing the large-intestine conditions, their amounts were reduced. The breakdown of astragalin, which contains kaempferol in its structure, was associated with an increase in the content of this particular flavonol during the digestion of the non-conditioned preparation.

In many cases, an initial increase in polyphenol content was observed due to its release from various leaf matrix structures [43]. In general, in the gastrointestinal tract, there is a gradual release of compounds from different structures and an improvement in their availability through the conversion to other compounds to enable their appropriate activity or improve absorption. Determination of the specific antioxidant activity of the digestive content at different stages of digestion using analytical tests may be considered for future research.

## 5. Conclusions

The results of the study provide a further argument for determining the appropriate dosage of mulberry leaf teas that will have a beneficial health-promoting effect for consumers. To date, infusion manufacturers provide quantities of the recommended intake but do not refer to any dietary guidelines or recommendations in this aspect. It is important to remember that people who drink mulberry leaf teas probably already use anti-diabetic medications. Given the reduction in antioxidant compounds observed in studies as a result of *in vitro* digestion, it could be expected that consumers would want to increase their intake of mulberry teas. However, we need to be highly cautious about introducing mulberry leaf infusions into the diet, bearing in mind possible interactions with medications.

The simple mulberry leaf conditioning process proposed here resulted in different transformations of polyphenols during *in vitro* digestion. Thus, with the above in mind, it seems reasonable to continue, in the future, the study on the bioavailability of bioactive components from mulberry leaves subjected to the proposed conditions in *in vivo* models.

## Figures and Tables

**Table 1:** Schedule of conditions stages occurring at the *in vitro* digestion simulation.

Digestion Stage	Actor	Action Time	pH
Stomach	Pepsin	2 h	2.0
Duodenum	Pancreatic extract + bile acids	0.5 h	6.0
Small intestine	Intestine microflora	2 h	7.4
Large intestine		18 h	8.0

**Table 2:** The correlation coefficient r between conditioning time and *in vitro* digestion stage and the content of individual phenolic acids in digested conditioned and non-conditioned *Morus alba* L. leaf preparations.

Phenolic Acids	Conditioning Process			Digestion Stage		
	Spearman Rank Order	Gamma	τ Kendall	Spearman Rank Order	Gamma	τ Kendall
GAL	0.111	0.065	0.058	0.201	0.173 *	0.159 *
PRO	0.612 *	0.539 *	0.485 *	−0.559 *	−0.469 *	−0.431 *
HYD	0.548 *	0.481 *	0.433 *	0.280 *	0.220 *	0.202 *
VAN	0.276 *	0.205 *	0.184 *	0.489 *	0.417 *	0.383 *
CHL	0.217 *	0.154	0.138	−0.582 *	−0.492 *	−0.452 *
CAF	0.249 *	0.198 *	0.178 *	0.167	0.129	0.119
SYR	0.132	0.101	0.089	−0.840 *	−0.760 *	−0.701 *
COU	0.152	0.138	0.124	−0.072	−0.054	−0.050
FER	0.022	−0.033	−0.030	−0.136	−0.121	−0.111
SIN	0.328 *	0.240 *	0.216 *	−0.391 *	−0.321 *	−0.295 *
sum	−0.601 *			−0.112		

GAL–gallic acid, PRO–protocatechuic acid, HYD–4-hydroxybenzoic acid, VAN–vanillic acid, CHL–chlorogenic acid, CAF–caffeic acid, SYR–syringic acid, COU–*p-*coumaric acid, FER–ferulic acid, SIN–sinapic acid; *p* ≤ 0.05 in non-parametric tests; * statistically significant coefficient.

**Table 3:** Correlation coefficients r between conditioning time, *in vitro* digestion stage, and flavonol content of digested conditioned and non-conditioned *Morus alba* L. leaf preparations.

Flavonol	Conditioning Process			Digestion Stage		
	Spearman Rank Order	Gamma	τ Kendall	Spearman Rank Order	Gamma	τ Kendall
RUT	0.022	−0.034	−0.031	−0.678 *	−0.575 *	−0.528 *
AST	0.273 *	0.244 *	0.219 *	−0.046	−0.051	−0.047
QUE	0.462 *	0.388 *	0.349 *	0.211 *	0.163 *	0.149 *
KEM	−0.022	−0.023	−0.021	−0.135	−0.102	−0.094
ISR	−0.002	0.017	0.015	−0.047 *	−0.374 *	−0.344
	Pearson					
ISQ	0.350 * *p* = 0.001			−0.433 * *p* = 0.000		
MAL	0.200 *p* = 0.059			−0.424 * *p* = 0.000		
MYR	0.058 *p* = 0.590			−0.305 * *p* = 0.003		
Sum	−0.541 *			−0.540 *		

RUT–rutin, AST–astragalin, QUE–quercetin, KEM–kaempferol, ISR—isorhamnetin, ISQ–isoquercitrin, MAL–quercetin 3-O-(6″-O-malonyl)-β-D-glucoside, MYR–myricetin; *p* ≤ 0.05 in non-parametric tests; * statistically significant coefficient.

**Table 4:** The correlation coefficient r between conditioning time and *in vitro* digestion stage and the content of individual phenolic acids in digested infusions of conditioned and non-conditioned *Morus alba* L. leaves.

Phenolic Acids	Conditioning Process			Digestion Stage		
	Spearman Rank Order	Gamma	τ Kendall	Spearman Rank Order	Gamma	τ Kendall
GAL	−0.201	−0.162 *	−0.145 *	0.767 *	0.661 *	0.607 *
PRO	−0.647 *	−0.565 *	−0.504 *	−0.186	−0.142	−0.129
HYD	−0.147	−0.089	−0.079	0.247 *	0.208 *	0.186 *
VAN	−0.117	−0.095	−0.083	0.127	0.106	0.093
CHL	−0.597 *	−0.496 *	−0.447 *	−0.449 *	−0.377 *	−0.346 *
CAF	−0.813 *	−0.760 *	−0.684 *	0.189	0.156 *	0.143 *
SYR	−0.458 *	−0.579 *	−0.375 *	−0.355 *	−0.462 *	−0.302 *
COU	−0.594 *	−0.503 *	−0.459 *	−0.192	−0.161 *	−0.147 *
FER	−0.818 *	−0.732 *	−0.666 *	−0.296 *	−0.241 *	−0.221 *
SIN	−0.499 *	−1.000 *	−0.437 *	−0.264 *	−0.521 *	−0.221 *
sum	−0.402 *			0.699 *		

GAL–gallic acid, PRO–protocatechuic acid, HYD–4-hydroxybenzoic acid, VAN–vanillic acid, CHL–chlorogenic acid, CAF–caffeic acid, SYR–syringic acid, COU–*p-*coumaric acid, FER–ferulic acid, SIN–sinapic acid; *p* ≤ 0.05 in non-parametric tests; * statistically significant coefficient.

**Table 5:** The correlation coefficient r between conditioning time and *in vitro* digestion stage and the content of individual flavonols in digested infusions of conditioned and non-conditioned *Morus alba* L. leaves.

Flavonol	Conditioning Process			Digestion Stage		
	Spearman Rank Order	Gamma	τ Kendall	Spearman Rank Order	Gamma	τ Kendall
RUT	−0.639 *	−0.550 *	−0.495 *	−0.463 *	−0.391 *	−0.359 *
KEM	−0.622 *	−0.521 *	−0.468 *	−0.366 *	−0.305 *	−0.281 *
ISQ	−0.435 *	−0.353 *	−0.318 *	−0.572 *	−0.477 *	−0.438 *
MAL	−0.551 *	−0.452 *	−0.406 *	−0.402 *	−0.340 *	−0.313 *
AST	−0.609 *	−0.537 *	−0.453 *	−0.399 *	−0.348 *	−0.319 *
MYR	−0.589 *	−0.470 *	−0.423 *	−0.319 *	−0.276 *	−0.253 *
QUE	−0.412 *	−0.333 *	−0.299 *	−0.458 *	−0.371 *	−0.341 *
sum		−0.567 *			−0.454 *	

RUT–rutin, KEM–kaempferol, ISQ–isoquercitrin, MAL–quercetin 3-O-(6″-*O*-malonyl)-β-D-glucoside, AST–astragalin, MYR–myricetin, QUE–quercetin; *p* ≤ 0.05 in non-parametric tests; * statistically significant coefficient.

**Table 6:** The correlation coefficient r between the indices obtained at different stages of *in vitro* digestion of *Morus alba* leaf preparations and infusions of *Morus alba* leaf preparations.

Test	Digested Preparations/Digested Infusions
flavonols	0.661 *
phenolic acids	0.094

* Correlation coefficient statistically significant at *p* ≤ 0.05.

## Data Availability

The datasets of the current study are available from the corresponding author upon reasonable request. The data are not publicly available due to Poznań University of Life Sciences privacy restrictions.

## Supplementary Materials

The following supporting information can be downloaded at: https://www.mdpi.com/article/10.3390/metabo14010031/s1, Table S1: Phenolic acid content at different stages of the simulated digestion process of conditioned and non-conditioned *Morus alba* L. leaf preparations [μg × mL^−1^]; Table S2: Flavonols content at different stages of the simulated digestion process of conditioned and non-conditioned *Morus alba* L. leaf preparations [μg × mL^−1^]; Table S3: Phenolic acid content at different stages of the simulated digestion process of infusions of conditioned and non-conditioned *Morus alba* L. leaves [μg × mL^−1^]; Table S4: Flavonols content at different stages of the simulated digestion process of infusions of conditioned and non-conditioned *Morus alba* L. leaves [μg × mL^−1^].
