# Supplementary material for: How Does In Vitro Digestion Change the Amount of Phenolics in Morus alba L. Leaf? Analysis of Preparations and Infusions
Source: Metabolites. 2024 Jan 1;14(1):31. doi: 10.3390/metabo14010031 (PMC10818460; doi:10.3390/metabo14010031)
Supplement: Supplementary file 1 [file metabolites-14-00031-s001.zip › metabolites-2777769-supplementary.pdf]

Table S1. Phenolic acid content at different stages of the simulated digestion process of conditioned and non-conditioned *Morus alba* L. leaf preparations [μg × mL $^{-1}$].

Phenolic acids [μg × mL^{-1}]			Stages of digestion process					
			A	B	C	D	E	F
0h	GAL		7.96	7.35	7.42	7.32	4.59	2.72
		SD	0.03	0.07	0.02	0.00	0.01	0.02
	PRO		0.59	0.54	0.46	0.41	0.35	0.13
		SD	0.00	0.00	0.00	0.00	0.00	0.00
	HYD		0.03	0.04	0.03	0.04	0.03	0.08
		SD	0.00	0.00	0.00	0.00	0.00	0.00
	VAN		0.03	0.04	0.05	0.07	0.07	0.09
		SD	0.00	0.00	0.00	0.00	0.00	0.00
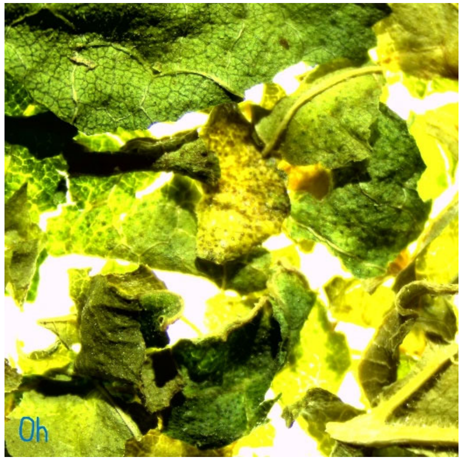	CHL		2.56	2.40	1.68	1.41	0.55	0.00
		SD	0.02	0.01	0.05	0.01	0.00	0.00
	CAF		2.18	1.88	1.80	1.40	0.78	0.58
		SD	0.02	0.00	0.03	0.02	0.00	0.00
	SYR		0.04	0.03	0.02	0.01	0.01	0.00
		SD	0.00	0.00	0.00	0.00	0.00	0.00
	COU		0.01	0.01	0.01	0.01	0.01	0.00
		SD	0.00	0.00	0.00	0.00	0.00	0.00
	FER		0.03	0.03	0.03	0.03	0.02	0.01
		SD	0.00	0.00	0.00	0.00	0.00	0.00
	SIN		0.02	0.01	0.01	0.02	0.01	0.00
		SD	0.00	0.00	0.00	0.00	0.00	0.00
1h	GAL		3.16	3.02	6.10	4.05	5.20	5.81
		SD	0.03	0.00	0.01	0.01	0.02	0.00

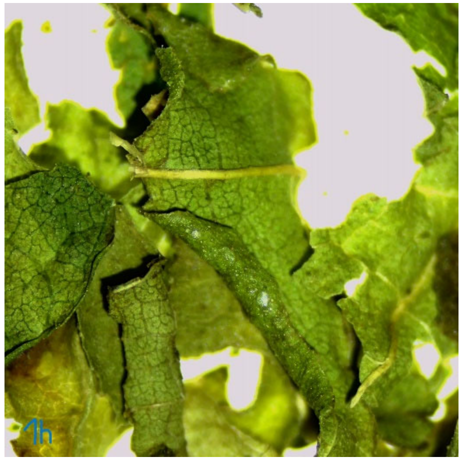	PRO		0.13	0.12	0.14	0.11	0.07	0.03
		SD	0.00	0.00	0.00	0.00	0.00	0.00
	HYD		0.00	0.00	0.02	0.01	0.01	0.04
		SD	0.00	0.00	0.00	0.00	0.00	0.00
	VAN		0.01	0.01	0.03	0.02	0.02	0.05
		SD	0.00	0.00	0.00	0.00	0.00	0.00
	CHL		1.18	0.96	1.00	0.32	0.37	0.10
		SD	0.00	0.00	0.04	0.00	0.00	0.00
	CAF		0.68	0.55	0.99	0.76	0.62	0.85
		SD	0.00	0.00	0.01	0.00	0.00	0.01
	SYR		0.03	0.02	0.01	0.00	0.00	0.00
		SD	0.00	0.00	0.00	0.00	0.00	0.00
	COU		0.00	0.00	0.00	0.00	0.01	0.01
		SD	0.00	0.00	0.00	0.00	0.00	0.00
	FER		0.02	0.02	0.03	0.02	0.02	0.02
		SD	0.00	0.00	0.00	0.00	0.00	0.00
	SIN		0.01	0.00	0.01	0.01	0.01	0.01
		SD	0.00	0.00	0.00	0.00	0.00	0.00
2h	GAL		2.12	2.69	2.98	3.12	4.42	4.81
		SD	0.01	0.02	0.02	0.01	0.03	0.04
	PRO		0.18	0.21	0.20	0.19	0.21	0.08
		SD	0.00	0.00	0.00	0.00	0.00	0.00
	HYD		0.01	0.02	0.01	0.02	0.00	0.03
		SD	0.00	0.00	0.00	0.00	0.00	0.00
	VAN		0.01	0.02	0.02	0.02	0.04	0.05
		SD	0.00	0.00	0.00	0.00	0.00	0.00
	CHL		0.91	1.16	1.03	0.90	1.14	0.43

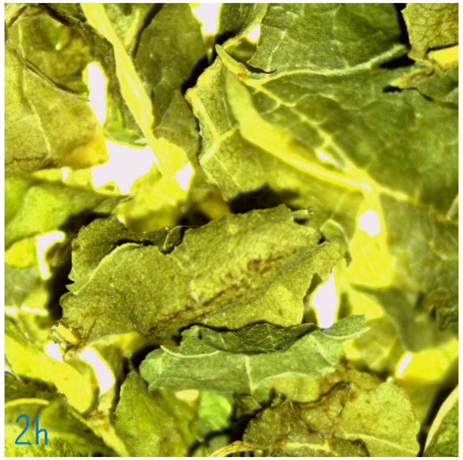		SD	0.01	0.01	0.02	0.00	0.00	0.00
	CAF		0.57	0.76	0.74	0.74	1.26	1.23
		SD	0.00	0.00	0.00	0.01	0.01	0.00
	SYR		0.01	0.01	0.02	0.03	0.00	0.00
		SD	0.00	0.00	0.00	0.00	0.00	0.00
	COU		0.00	0.00	0.01	0.01	0.00	0.00
		SD	0.00	0.00	0.00	0.00	0.00	0.00
	FER		0.01	0.01	0.01	0.01	0.02	0.01
		SD	0.00	0.00	0.00	0.00	0.00	0.00
	SIN		0.01	0.00	0.00	0.01	0.00	0.01
		SD	0.00	0.00	0.00	0.00	0.00	0.00
3h 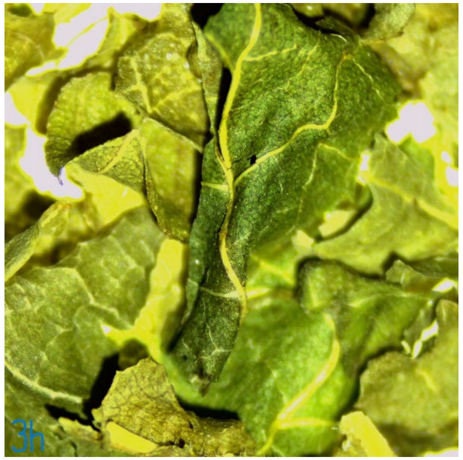	GAL		2.08	2.98	3.13	3.68	2.86	2.35
		SD	0.00	0.01	0.02	0.00	0.01	0.01
	PRO		0.24	0.26	0.25	0.10	0.06	0.04
		SD	0.00	0.00	0.01	0.00	0.00	0.00
	HYD		0.01	0.02	0.03	0.03	0.02	0.04
		SD	0.00	0.00	0.00	0.00	0.00	0.00
	VAN		0.00	0.03	0.02	0.03	0.03	0.04
		SD	0.00	0.00	0.00	0.00	0.00	0.00
	CHL		0.97	1.24	1.00	0.75	0.76	0.22
		SD	0.00	0.00	0.04	0.00	0.00	0.00
	CAF		0.66	0.90	0.87	1.06	1.03	1.41
		SD	0.00	0.00	0.00	0.00	0.00	0.02
	SYR		0.01	0.03	0.01	0.01	0.01	0.00
		SD	0.00	0.00	0.00	0.00	0.00	0.00
	COU		0.00	0.00	0.01	0.01	0.01	0.00
		SD	0.00	0.00	0.00	0.00	0.00	0.00

	FER		0.01	0.02	0.01	0.02	0.01	0.01
		SD	0.00	0.00	0.00	0.00	0.00	0.00
	SIN		0.01	0.01	0.00	0.01	0.00	0.00
		SD	0.00	0.00	0.00	0.00	0.00	0.00
4h	GAL		2.43	3.29	2.79	3.09	3.09	4.21
		SD	0.00	0.07	0.01	0.02	0.02	0.01
	PRO		0.23	0.28	0.20	0.22	0.20	0.05
		SD	0.00	0.00	0.00	0.00	0.00	0.00
	HYD		0.02	0.03	0.02	0.02	0.02	0.01
		SD	0.00	0.00	0.00	0.00	0.00	0.00
	VAN		0.02	0.02	0.02	0.02	0.02	0.01
		SD	0.00	0.00	0.00	0.00	0.00	0.00
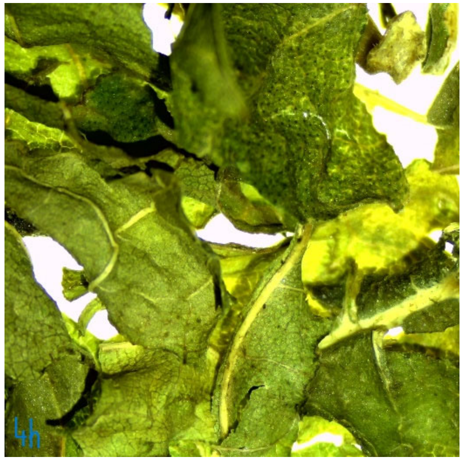	CHL		0.71	0.98	0.77	0.72	0.62	1.07
		SD	0.00	0.00	0.04	0.01	0.00	0.00
	CAF		0.60	0.83	0.55	0.63	0.56	0.97
		SD	0.01	0.00	0.00	0.00	0.00	0.00
	SYR		0.02	0.02	0.01	0.01	0.00	0.00
		SD	0.00	0.00	0.00	0.00	0.00	0.00
	COU		0.01	0.01	0.00	0.00	0.00	0.00
		SD	0.00	0.00	0.00	0.00	0.00	0.00
	FER		0.01	0.01	0.01	0.01	0.01	0.01
		SD	0.00	0.00	0.00	0.00	0.00	0.00
	SIN		0.01	0.01	0.01	0.01	0.00	0.00
		SD	0.00	0.00	0.00	0.00	0.00	0.00

A – sampling at the beginning of stomach stage, B – sampling at the end of stomach stage, C – sampling at the end of duodenum stage, D – sampling at the beginning of small intestine, E – sampling at the end of small intestine, F - sampling at the end of large intestine; GAL - gallic acid, PRO - protocatechuic acid, HYD - 4-hydroxybenzoic acid, VAN - vanillic acid, CHL - chlorogenic acid, CAF - caffeic acid, SYR - syringic acid, COU - *p*-coumaric acid, FER - ferulic acid, SIN - sinapic acid; 0h - *Morus alba* L. leaves non-conditioned, 1h – *Morus alba* L. leaves conditioned for 1 hour, 2h – *Morus alba* L. leaves conditioned for 2 hours, 3h – *Morus alba* L. leaves conditioned for 3 hours, 4h – *Morus alba* L. leaves conditioned for 4 hours; SD – standard deviation.

Table S2. Flavonols content at different stages of the simulated digestion process of conditioned and non-conditioned *Morus alba* L. leaf preparations [μg × mL $^{-1}$].

Flavonols [μg × mL $^{-1}$]			Stages of digestion process					
			A	B	C	D	E	F
0h	RUT		80.81	68.86	53.33	32.94	48.61	17.66
		SD	0.01	1.01	3.07	1.85	1.28	4.28
	ISQ		17.63	13.42	15.12	9.11	14.65	8.66
		SD	0.03	0.28	0.99	0.74	0.38	1.27
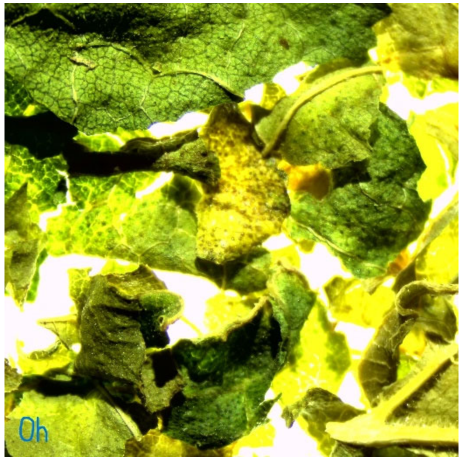	MAL		78.65	54.54	46.00	33.84	43.81	22.35
		SD	0.05	0.40	2.23	2.77	0.50	0.45
	AST		5.41	4.16	6.00	5.34	8.24	5.58
		SD	0.02	0.25	1.06	0.65	0.98	0.75
	MYR		12.37	10.37	14.30	11.31	11.21	0.73
		SD	0.03	0.19	0.23	0.31	0.40	0.19
	QUE		0.24	0.23	0.23	0.38	0.23	0.14
		SD	0.00	0.00	0.01	0.02	0.00	0.01
	KEM		0.18	0.15	0.22	0.18	0.19	0.19
		SD	0.00	0.01	0.05	0.01	0.01	0.06
	ISR		0.60	0.60	0.61	0.57	0.56	0.52
		SD	0.01	0.02	0.07	0.01	0.00	0.00
1h	RUT		52.95	51.65	49.86	46.76	34.62	20.63
		SD	0.43	0.05	1.56	0.31	0.35	6.75
	ISQ		19.37	19.82	22.89	22.03	15.82	7.02
		SD	0.45	0.05	0.10	0.37	0.18	1.55
	MAL		55.40	53.69	65.57	75.05	54.35	34.95
		SD	0.14	0.07	0.25	1.62	0.76	2.75

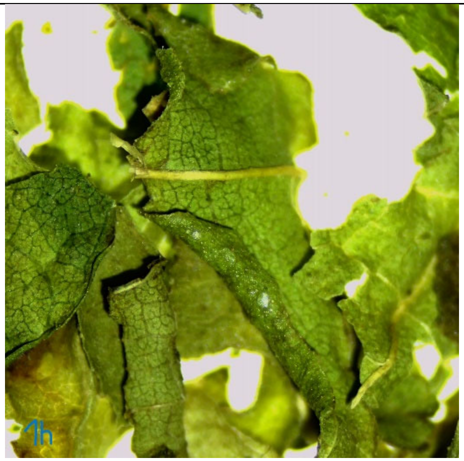	AST		6.01	7.92	8.57	8.57	8.11	5.85
		SD	0.33	0.06	0.25	0.16	0.05	0.27
	MYR		9.45	9.20	11.51	13.83	11.12	7.38
		SD	0.13	0.07	0.11	0.42	0.09	1.99
	QUE		0.33	0.44	0.29	0.36	0.34	0.26
		SD	0.02	0.01	0.00	0.01	0.00	0.05
	KEM		0.48	0.43	0.24	0.17	0.17	0.18
		SD	0.02	0.01	0.02	0.01	0.01	0.04
	ISR		0.66	0.61	0.65	0.56	0.58	0.55
		SD	0.01	0.05	0.05	0.00	0.01	0.01
2h	RUT		49.37	48.71	44.31	36.90	35.49	2.62
		SD	0.11	1.11	0.19	1.03	0.58	0.14
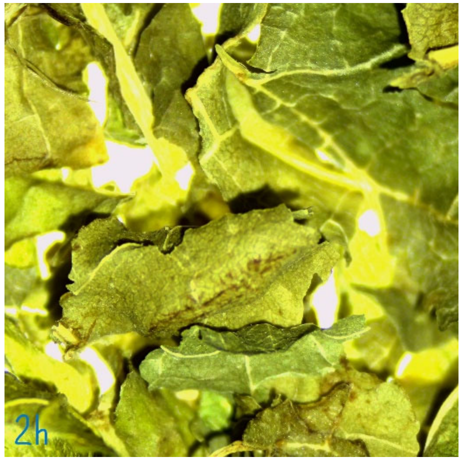	ISQ		20.13	24.67	25.46	21.27	21.73	5.25
		SD	0.17	0.01	0.25	0.92	0.77	1.69
	MAL		42.53	56.04	63.98	52.58	52.75	31.16
		SD	0.07	0.02	0.40	2.39	1.30	0.22
	AST		7.03	8.82	8.76	8.95	9.76	7.84
		SD	0.04	0.04	0.07	0.19	0.68	0.49
	MYR		6.98	9.50	11.27	9.98	9.99	3.35
		SD	0.05	0.07	0.12	0.28	0.12	1.03
	QUE		0.26	0.55	0.46	0.93	0.96	0.29
		SD	0.01	0.03	0.13	0.04	0.01	0.01
	KEM		0.38	0.25	0.27	0.33	0.35	0.16
		SD	0.01	0.02	0.00	0.02	0.00	0.01
	ISR		0.66	0.66	0.78	0.68	0.57	0.61
		SD	0.14	0.03	0.00	0.04	0.01	0.02
3h	RUT		33.39	39.12	25.91	26.83	33.60	11.51
		SD	0.49	0.24	0.03	0.82	4.46	0.19

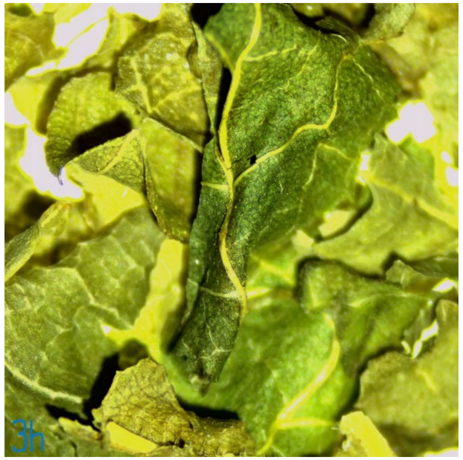	ISQ		11.00	14.63	11.58	12.25	17.00	18.39
		SD	0.09	0.03	0.03	0.04	1.34	0.19
	MAL		30.61	37.40	31.26	35.29	44.18	24.75
		SD	0.07	0.05	0.01	0.04	1.27	0.14
	AST		4.58	6.23	5.06	5.14	4.79	6.86
		SD	0.12	0.01	0.01	0.04	0.10	0.09
	MYR		5.35	6.44	5.49	6.26	8.00	2.43
		SD	0.09	0.01	0.01	0.00	1.01	0.96
	QUE		0.26	0.31	0.33	0.27	0.32	0.58
		SD	0.00	0.01	0.00	0.01	0.02	0.02
	KEM		0.16	0.18	0.19	0.16	0.17	0.20
		SD	0.00	0.00	0.00	0.00	0.01	0.01
	ISR		0.58	0.59	0.57	0.56	0.55	0.55
		SD	0.00	0.00	0.00	0.00	0.00	0.02
4h 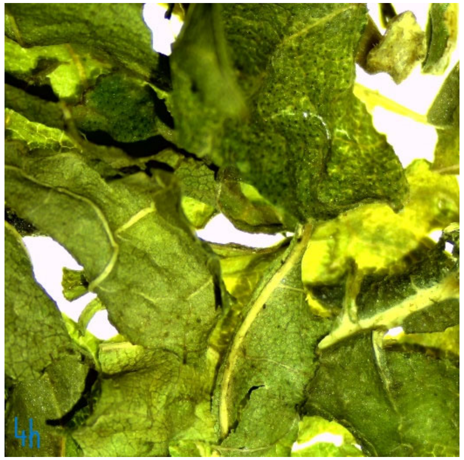	RUT		103.88	100.08	84.95	76.84	51.01	0.68
		SD	0.39	0.16	4.80	0.24	0.49	0.12
	ISQ		32.60	29.91	30.33	32.30	21.09	1.28
		SD	0.93	1.64	2.18	1.32	1.30	0.55
	MAL		82.12	71.73	83.36	92.47	61.34	41.88
		SD	0.21	0.06	1.81	1.11	2.90	0.15
	AST		11.98	11.10	11.20	12.87	6.93	5.07
		SD	0.16	0.08	1.31	0.66	0.54	0.16
	MYR		13.92	13.90	15.00	19.86	15.70	1.61
		SD	0.09	0.08	3.19	0.63	3.12	0.31
	QUE		0.33	0.30	0.37	0.82	0.81	0.63
		SD	0.00	0.00	0.08	0.02	0.06	0.03
	KEM		0.18	0.17	0.19	0.32	0.41	0.16
		SD	0.02	0.00	0.02	0.03	0.09	0.01
	ISR		0.58	0.58	0.59	0.60	0.58	0.58
		SD	0.04	0.00	0.01	0.01	0.01	0.01

A – sampling at the beginning of stomach stage, B – sampling at the end of stomach stage, C – sampling at the end of duodenum stage, D – sampling at the beginning of small intestine, E – sampling at the end of small intestine, F - sampling at the end of large intestine; RUT - rutin, ISQ - isoquercitrin, MAL - quercetin 3-O-(6”-O-malonyl)-β-D-glucoside, AST - astragalin, MYR – myricetin, QUE – quercetin, KEM - kaempferol, ISR – isorhamnetin; 0h - *Morus alba* L. leaves non-conditioned, 1h – *Morus alba* L. leaves conditioned for 1 hour, 2h – *Morus alba* L. leaves conditioned for 2 hours, 3h – *Morus alba* L. leaves conditioned for 3 hours, 4h – *Morus alba* L. leaves conditioned for 4 hours.; SD – standard deviation.

Table S3. Phenolic acid content at different stages of the simulated digestion process of infusions of conditioned and non-conditioned *Morus alba* L. leaves [μg × mL $^{-1}$].

Phenolic acids [μg × mL $^{-1}$]		Stages of digestion process					
		A	B	C	D	E	F
0N 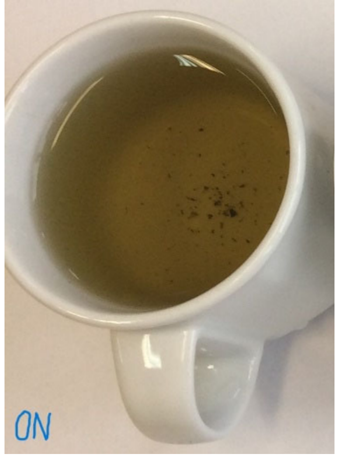	GAL	0.60	0.73	1.05	2.68	2.31	2.74
	SD	0.00	0.00	0.00	0.02	0.02	0.00
	PRO	0.07	0.09	0.10	0.14	0.10	0.02
	SD	0.00	0.00	0.00	0.01	0.00	0.00
	HYD	0.00	0.00	0.00	0.00	0.00	0.01
	SD	0.00	0.00	0.00	0.00	0.00	0.00
	VAN	0.00	0.00	0.00	0.00	0.01	0.02
	SD	0.00	0.00	0.00	0.00	0.00	0.00
	CHL	0.34	0.41	0.36	0.26	0.24	0.09
	SD	0.01	0.01	0.00	0.00	0.00	0.00
	CAF	0.52	0.61	0.56	0.55	0.52	0.51
	SD	0.01	0.00	0.01	0.02	0.01	0.02
	SYR	0.00	0.00	0.01	0.00	0.00	0.00
	SD	0.00	0.00	0.00	0.00	0.00	0.00
	COU	0.01	0.01	0.01	0.01	0.01	0.00
	SD	0.00	0.00	0.00	0.00	0.00	0.00
	FER	0.01	0.01	0.01	0.01	0.01	0.01
	SD	0.00	0.00	0.00	0.00	0.00	0.00
	SIN	0.00	0.00	0.01	0.00	0.00	0.00
	SD	0.00	0.00	0.00	0.00	0.00	0.00
1N	GAL	1.26	0.55	1.15	2.04	2.14	2.05
	SD	0.01	0.01	0.01	0.00	0.00	0.01
	PRO	0.05	0.01	0.07	0.06	0.08	0.03
	SD	0.00	0.00	0.00	0.00	0.00	0.00
	HYD	0.00	0.00	0.00	0.00	0.00	0.00
	SD	0.00	0.00	0.00	0.00	0.00	0.00
	VAN	0.00	0.00	0.00	0.00	0.00	0.00

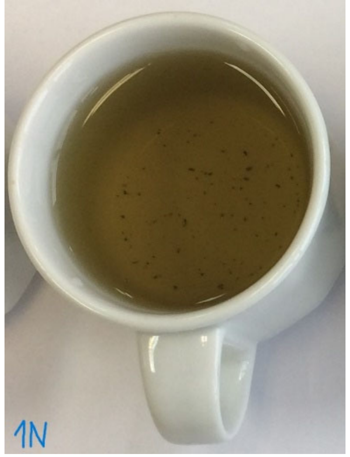	SD	0.00	0.00	0.00	0.00	0.00	0.00
	CHL	0.40	0.20	0.42	0.29	0.32	0.19
	SD	0.01	0.01	0.00	0.00	0.01	0.00
	CAF	0.42	0.20	0.46	0.45	0.45	0.58
	SD	0.00	0.00	0.02	0.00	0.02	0.03
	SYR	0.01	0.01	0.01	0.00	0.00	0.00
	SD	0.00	0.00	0.00	0.00	0.00	0.00
	COU	0.01	0.00	0.00	0.00	0.00	0.00
	SD	0.00	0.00	0.00	0.00	0.00	0.00
	FER	0.01	0.01	0.01	0.01	0.01	0.01
	SD	0.00	0.00	0.00	0.00	0.00	0.00
	SIN	0.00	0.00	0.00	0.00	0.00	0.00
	SD	0.00	0.00	0.00	0.00	0.00	0.00
2N 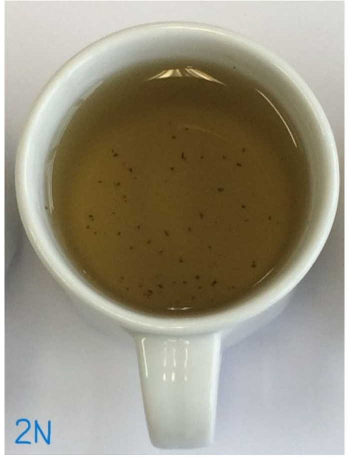	GAL	0.19	0.85	1.24	1.85	2.48	2.24
	SD	0.00	0.01	0.01	0.01	0.14	0.08
	PRO	0.00	0.07	0.10	0.01	0.01	0.00
	SD	0.00	0.01	0.00	0.00	0.00	0.00
	HYD	0.00	0.00	0.00	0.00	0.00	0.01
	SD	0.00	0.00	0.00	0.00	0.00	0.00
	VAN	0.00	0.00	0.01	0.00	0.00	0.00
	SD	0.00	0.00	0.00	0.00	0.00	0.00
	CHL	0.12	0.34	0.24	0.11	0.11	0.05
	SD	0.00	0.01	0.00	0.00	0.00	0.00
	CAF	0.12	0.42	0.80	0.42	0.51	0.35
	SD	0.00	0.00	0.00	0.01	0.01	0.01
	SYR	0.00	0.00	0.00	0.00	0.00	0.00
	SD	0.00	0.00	0.00	0.00	0.00	0.00
	COU	0.00	0.00	0.01	0.00	0.01	0.00
	SD	0.00	0.00	0.00	0.00	0.00	0.00
	FER	0.01	0.01	0.01	0.01	0.01	0.01
	SD	0.00	0.00	0.00	0.00	0.00	0.00
	SIN	0.00	0.00	0.00	0.00	0.00	0.00
	SD	0.00	0.00	0.00	0.00	0.00	0.00

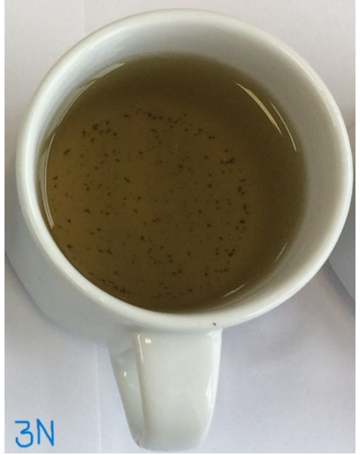

3N	GAL	0.20	0.73	1.04	2.34	2.41	3.49
	SD	0.01	0.01	0.04	0.03	0.05	0.10
	PRO	0.06	0.06	0.07	0.01	0.03	0.00
	SD	0.00	0.00	0.00	0.00	0.00	0.00
	HYD	0.01	0.01	0.00	0.01	0.01	0.01
	SD	0.00	0.00	0.00	0.00	0.00	0.00
	VAN	0.00	0.00	0.00	0.00	0.00	0.01
	SD	0.00	0.00	0.00	0.00	0.00	0.00
	CHL	0.25	0.26	0.22	0.13	0.12	0.07
	SD	0.00	0.00	0.00	0.00	0.00	0.00
	CAF	0.26	0.28	0.30	0.27	0.29	0.39
	SD	0.00	0.01	0.01	0.00	0.00	0.01
	SYR	0.00	0.00	0.00	0.00	0.00	0.00
	SD	0.00	0.00	0.00	0.00	0.00	0.00
	COU	0.00	0.00	0.00	0.00	0.00	0.00
	SD	0.00	0.00	0.00	0.00	0.00	0.00
	FER	0.01	0.01	0.01	0.00	0.01	0.01
	SD	0.00	0.00	0.00	0.00	0.00	0.00
	SIN	0.00	0.00	0.00	0.00	0.00	0.00
	SD	0.00	0.00	0.00	0.00	0.00	0.00
4N	GAL	0.00	0.31	0.18	2.43	2.31	0.79
	SD	0.00	0.01	0.01	0.02	0.03	0.00
	PRO	0.00	0.02	0.00	0.00	0.01	0.00
	SD	0.00	0.00	0.00	0.00	0.00	0.00
	HYD	0.00	0.00	0.00	0.00	0.00	0.00
	SD	0.00	0.00	0.00	0.00	0.00	0.00
	VAN	0.00	0.00	0.00	0.00	0.01	0.01
	SD	0.00	0.00	0.00	0.00	0.00	0.00
	CHL	0.02	0.17	0.06	0.13	0.12	0.01
	SD	0.00	0.00	0.00	0.00	0.00	0.00
	CAF	0.02	0.16	0.06	0.20	0.21	0.05
	SD	0.00	0.01	0.00	0.00	0.00	0.00

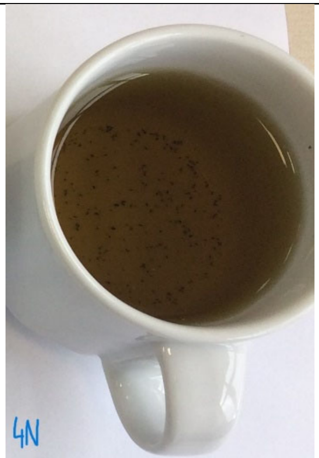

SYR	0.00	0.00	0.00	0.00	0.00	0.00
SD	0.00	0.00	0.00	0.00	0.00	0.00
COU	0.00	0.00	0.00	0.00	0.00	0.00
SD	0.00	0.00	0.00	0.00	0.00	0.00
FER	0.00	0.01	0.00	0.00	0.00	0.00
SD	0.00	0.00	0.00	0.00	0.00	0.00
SIN	0.00	0.00	0.00	0.00	0.00	0.00
	0.00	0.00	0.00	0.00	0.00	0.00
SD						

Table S4. Flavonols content at different stages of the simulated digestion process of infusions of conditioned and non-conditioned *Morus alba* L. leaves [µg × mL $^{-1}$].

Flavonols [µg × mL $^{-1}$]			Stages of digestion process					
			A	B	C	D	E	F
0N 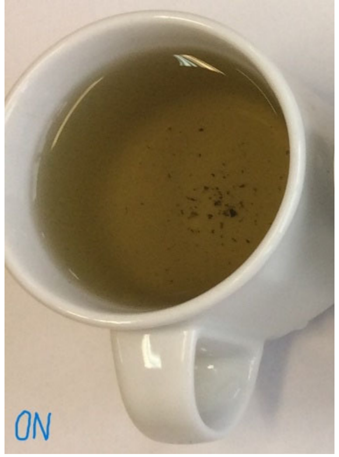	RUT		14.62	16.53	13.93	9.32	7.83	7.01
		SD	0.12	0.39	0.10	0.09	0.13	0.08
	KEM		0.28	0.24	0.24	0.24	0.21	0.21
		SD	0.01	0.02	0.00	0.00	0.01	0.00
	ISQ		5.87	6.38	5.76	3.86	3.18	0.27
		SD	0.20	0.10	0.02	0.02	0.06	0.00
	MAL		14.81	16.70	16.61	11.52	9.48	6.76
		SD	0.67	0.32	0.23	0.25	0.11	0.18
	AST		1.88	1.88	1.65	1.35	1.21	0.64
		SD	0.10	0.06	0.05	0.04	0.00	0.01
	MYR		2.05	2.26	2.30	1.75	1.42	1.10
		SD	0.12	0.03	0.02	0.02	0.02	0.03
	QUE		0.53	0.30	0.28	0.28	0.24	0.24
		SD	0.04	0.00	0.00	0.01	0.00	0.00
	ISR		0.00	0.00	0.00	0.00	0.00	0.00
		SD	0.00	0.00	0.00	0.00	0.00	0.00
1N	RUT		13.87	10.53	12.41	7.76	7.76	10.27
		SD	2.07	0.39	0.03	0.15	0.08	0.22
	KEM		0.33	0.25	0.21	0.19	0.20	0.19
		SD	0.02	0.01	0.01	0.02	0.00	0.01
	ISQ		7.52	6.11	7.28	2.44	1.74	3.92
		SD	0.22	0.19	0.09	0.03	0.01	0.08
	MAL		14.77	9.74	14.79	12.65	14.84	13.58
		SD	0.32	0.19	0.34	0.05	0.09	0.35
	AST		1.60	1.91	1.63	0.85	0.93	0.98
		SD	0.14	0.09	0.08	0.07	0.05	0.05
	MYR		1.92	1.40	2.48	2.13	2.27	1.94

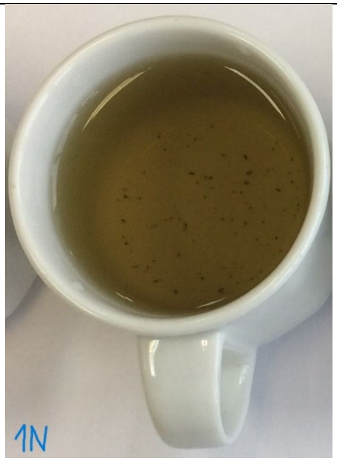		SD	0.08	0.07	0.07	0.04	0.06	0.03
	QUE		0.31	0.43	0.26	0.59	0.72	0.27
		SD	0.06	0.01	0.01	0.05	0.06	0.00
	ISR		0.00	0.00	0.00	0.00	0.00	0.00
			0.00	0.00	0.00	0.00	0.00	0.00
		SD						
2N	RUT		5.61	18.09	15.74	6.49	7.33	4.88
		SD	0.05	0.06	0.09	0.01	0.08	0.16
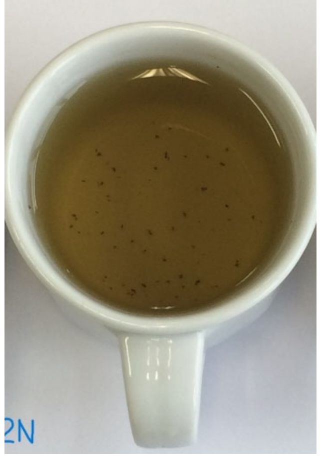	KEM		0.18	0.32	0.22	0.18	0.19	0.17
		SD	0.00	0.01	0.00	0.00	0.01	0.00
	ISQ		2.69	8.97	10.19	3.36	4.54	0.22
		SD	0.06	0.09	0.05	0.02	0.05	0.01
	MAL		6.93	20.34	16.72	6.61	6.91	3.43
		SD	0.05	0.08	0.46	0.05	0.11	0.03
	AST		0.51	1.39	1.77	0.69	0.69	0.42
		SD	0.02	0.01	0.01	0.03	0.01	0.01
	MYR		0.95	2.73	2.55	1.11	1.19	0.69
		SD	0.01	0.04	0.02	0.03	0.03	0.03
	QUE		0.36	0.69	0.35	0.27	0.26	0.23
		SD	0.00	0.02	0.01	0.01	0.00	0.00
	ISR		0.00	0.00	0.00	0.00	0.00	0.00
		SD	0.00	0.00	0.00	0.00	0.00	0.00
3N	RUT		10.16	10.16	8.81	5.53	5.59	4.77
		SD	0.16	0.14	0.24	0.11	0.03	0.10
	KEM		0.21	0.19	0.19	0.17	0.17	0.21
		SD	0.01	0.01	0.01	0.00	0.01	0.01

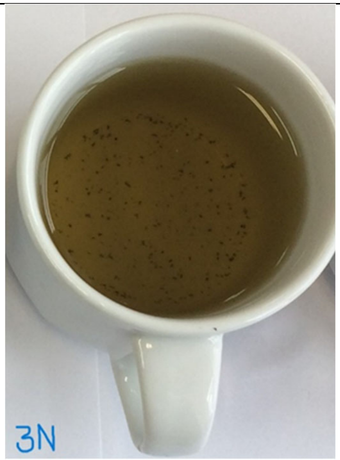	ISQ		4.39	4.19	3.91	2.58	2.51	0.16
		SD	0.08	0.19	0.09	0.07	0.17	0.01
	MAL		11.99	13.12	12.71	7.60	7.97	3.81
		SD	0.06	0.07	0.06	0.05	0.62	0.04
	AST		0.84	0.82	0.74	0.51	0.66	0.42
		SD	0.01	0.07	0.05	0.01	0.07	0.01
	MYR		1.76	1.73	1.72	1.14	1.08	0.74
		SD	0.03	0.03	0.04	0.02	0.04	0.01
	QUE		0.28	0.28	0.23	0.22	0.23	0.24
		SD	0.00	0.00	0.00	0.00	0.00	0.00
	ISR		0.00	0.00	0.00	0.00	0.00	0.00
		SD	0.00	0.00	0.00	0.00	0.00	0.00
4N	RUT		2.49	7.44	2.64	5.18	5.15	0.95
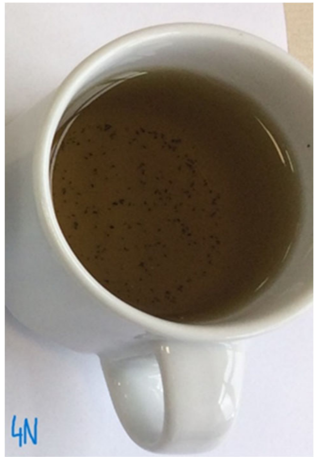		SD	0.05	0.03	0.04	0.09	0.02	0.05
	KEM		0.13	0.21	0.16	0.18	0.19	0.15
		SD	0.00	0.00	0.01	0.01	0.01	0.00
	ISQ		0.86	3.18	1.06	2.47	2.30	0.03
		SD	0.05	0.02	0.02	0.09	0.01	0.02
	MAL		1.34	8.62	3.49	7.39	6.82	0.83
		SD	0.02	0.07	0.06	0.18	0.10	0.06
	AST		0.23	1.10	0.41	0.88	0.82	0.18
		SD	0.01	0.00	0.01	0.04	0.07	0.02
	MYR		0.32	1.31	0.59	1.15	1.05	0.26
		SD	0.01	0.00	0.01	0.04	0.00	0.01
	QUE		0.24	0.30	0.22	0.28	0.26	0.22
		SD	0.01	0.06	0.00	0.01	0.01	0.00
	ISR		0.00	0.00	0.00	0.00	0.00	0.00
		SD	0.00	0.00	0.00	0.00	0.00	0.00
